# Insight into the Spatial Arrangement of the Lysine Tyrosylquinone and Cu^2+^ in the Active Site of Lysyl Oxidase-like 2

**DOI:** 10.3390/ijms232213966

**Published:** 2022-11-12

**Authors:** Alex A. Meier, Hee-Jung Moon, Sinan Sabuncu, Priya Singh, Trey A. Ronnebaum, Siyu Ou, Justin T. Douglas, Timothy A. Jackson, Pierre Moënne-Loccoz, Minae Mure

**Affiliations:** 1 Department of Chemistry, The University of Kansas, Lawrence, KS 66045, USA; 2 Department of Chemical Physiology and Biochemistry, School of Medicine, Oregon Health & Science University, Portland, OR 97239, USA

**Keywords:** lysyl oxidase-like 2, lysine tyrosylquinone, UV-vis spectroscopy, resonance Raman spectroscopy, model chemistry

## Abstract

Lysyl oxidase-2 (LOXL2) is a Cu^2+^ and lysine tyrosylquinone (LTQ)-dependent amine oxidase that catalyzes the oxidative deamination of peptidyl lysine and hydroxylysine residues to promote crosslinking of extracellular matrix proteins. LTQ is post-translationally derived from Lys653 and Tyr689, but its biogenesis mechanism remains still elusive. A 2.4 Å Zn^2+^-bound precursor structure lacking LTQ (PDB:5ZE3) has become available, where Lys653 and Tyr689 are 16.6 Å apart, thus a substantial conformational rearrangement is expected to take place for LTQ biogenesis. However, we have recently shown that the overall structures of the precursor (no LTQ) and the mature (LTQ-containing) LOXL2s are very similar and disulfide bonds are conserved. In this study, we aim to gain insights into the spatial arrangement of LTQ and the active site Cu^2+^ in the mature LOXL2 using a recombinant LOXL2 that is inhibited by 2-hydrazinopyridine (2HP). Comparative UV-vis and resonance Raman spectroscopic studies of the 2HP-inhibited LOXL2 and the corresponding model compounds and an EPR study of the latter support that 2HP-modified LTQ serves as a tridentate ligand to the active site Cu^2^. We propose that LTQ resides within 2.9 Å of the active site of Cu^2+^ in the mature LOXL2, and both LTQ and Cu^2+^ are solvent-exposed.

## 1. Introduction

**Figure 1:**
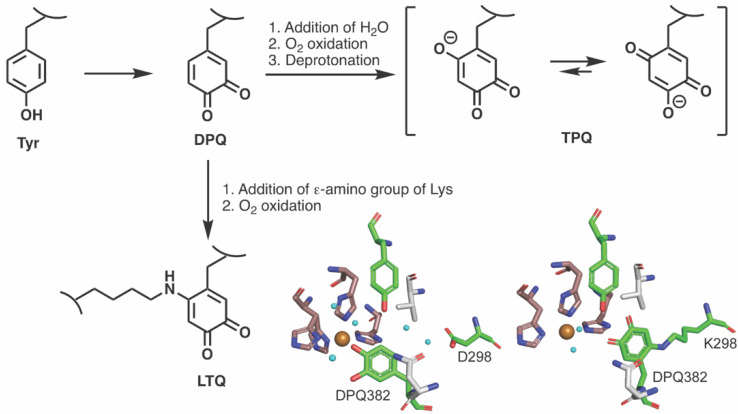
DPQ is proposed as a common intermediate in the biogenesis of TPQ and LTQ. 1,4−Hydration or 1,4−amination of DPQ followed by O_2_ oxidation yield TPQ or LTQ. DPQ detected during biogenesis of TPQ in AGAO by X−ray snapshot analysis [7] and an LTQ−like quinone (K298 crosslinked to DPQ382) detected in D298K−AGAO [10] are also shown. DPQ382, Tyr284 (conserved Tyr) and Asp298 (active site base): in green; Cu^2+^ binding site (His431−X−His433-----His592): in brown; Val282 and Asn381 (walls of the wedge identified in CAOs [11,12]): in gray.

Lysyl oxidase−like 2 (LOXL2) plays an essential role in remodeling of the extracellular matrix (ECM) by catalyzing the oxidative deamination of ϵ−amino group of lysine and hydroxylysine residues of ECM proteins such as collagens and tropoelastin (soluble precursor of elastin) to initiate their non−enzymatic crosslinking [1,2]. LOXL2 belongs to the lysyl oxidase (LOX)−family of proteins that are Cu^2+^−dependent amine oxidases (CAOs) but are distinct from other CAOs by having lysine tyrosylquinone (LTQ) as its organic cofactor instead of topaquinone (TPQ) [3,4]. The biogenesis mechanism of TPQ in CAOs has been extensively studied and it is autocatalytic requiring Cu^2+^ and oxygen [5,6,7,8,9]. An X−ray snapshot analysis of reaction intermediates during TPQ biogenesis in a bacterial CAO from *Arthrobacter globiformis* (AGAO) has indicated the presence of dopaquinone (DPQ) intermediate derived from a conserved Tyr residue in the active site [7]. Although the biogenesis mechanism of LTQ still remains elusive due to the lack of recombinant protein suitable to conduct mechanistic study, we have generated an LTQ−like quinone in a mutant form of AGAO where a lysine residue has been incorporated by site−directed mutagenesis (D298K−AGAO) [10]. This study strongly supports the common intermediacy of DPQ in TPQ and LTQ biogenesis, where hydration or amination (ε−amino group of Lys) of DPQ yields TPQ or LTQ, respectively (Figure 1). 

In 2018, Zhang et al. reported an X−ray crystal structure of a Zn^2+^−bound precursor form (PDB: 5ZE3) of a N-terminally truncated LOXL2 at 2.4 Å resolution [13]. This is a major breakthrough in the research field. However, this structure does not contain LTQ and the precursor residues (Lys653 and Tyr687) of LTQ were detected 16.6 Å apart. Subsequently, Vallet et al. generated the amine oxidase domain of a mature LOX structure (containing LTQ) by molecular modeling [14]. Interestingly, this 3D−modeled structure of LOX contains disulfide bonds with a different pattern of cystine−pairing from the Zn^2+^−bound precursor form [13]. The different pattern of cystine−pairing in the 3D−modeled LOX was based on experimental data of the native LOX isolated from a bovine aorta [15]. LTQ detected in D298K−AGAO [10] (the only structure available in PDB that contains LTQ) was successfully modeled in this structure, however modeling LTQ into the active site of LOX structure bearing the same pattern of cystine−pairing of the precursor LOXL2 was unsuccessful [14]. In both studies, it was proposed that a substantial conformational rearrangement including disulfide shuffling is required for LTQ biogenesis. 

Our previous work has focused on characterizing the extent of post−translational modifications of LOXL2 and defining the effect of PTMs on the biochemical/biophysical and biological functions of LOXL2 [16,17,18,19]. The LTQ cofactor contains an orthoquinone moiety and the C5 carbonyl group can be covalently modified by hydrazine (−NHNH_2_) derivatives [20,21]. The phenylhydrazine (PH)−modified LTQ containing peptide and LTQ precursor residues (Lys653 and Tyr689) were identified in a recombinant LOXL2 by mass−spectrometry based peptide mapping [16]. We have succeeded in producing a catalytically−competent (LTQ−containing, mature) recombinant LOXL2 in high purity using the FreeStyle™ 293 Expression System, and determined the hydrodynamic radii and radii of gyration of both the mature and the precursor LOXL2s to be very similar [22]. This suggests that the overall structures of LOXL2 before and after LTQ biogenesis are unaltered. By mass spectrometry−based disulfide−mapping of the mature and the precursor LOXL2s, the cysteine−pairing pattern of five disulfide bonds in the active site are found to be totally conserved [23]. These results indicate that the proposed substantial conformational rearrangement is less likely to take place during LTQ biogenesis. In this study, we wish to gain insight into the active site environment of the mature LOXL2, especially the spatial arrangement of LTQ and Cu^2+^, and so we conducted a comparative UV−vis and resonance Raman spectroscopic study of 2−hydrazinopyridine (2HP)−inhibited LOXL2 and corresponding model compounds, as well as an EPR spectroscopic study of the latter. 2HP is a derivative of PH and it has more stability over PH at physiological pH to not produce radical species [24,25]. 

## 2. Results

### 2.1. UV−Vis Spectroscopy of 2HP−Inhibited LOXL2

**Figure 2:**
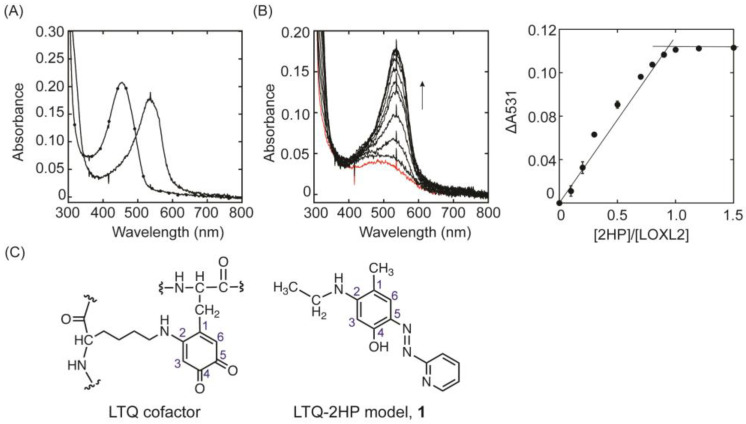
Inhibition of LOXL2 with 2HP. (**A**) UV−vis spectra of PH−inhibited LOXL2 (−−−) and 2HP−inhibited LOXL2 (−●−). (**B**) UV−vis spectroscopic titration of LTQ with 2HP. Increase in absorbance at 531 nm was observed. The UV-vis spectrum of the resting LOXL2 (containing unmodified LTQ) before addition of 2−HP is shown in red (left). The absorbance change at 531 nm (Δ531) was plotted against the molar ratio of [2HP]/[LOXL2]. The increase in absorption at 531 nm was saturated near [2HP]/[LOXL2] = 1.0 (right). The UV−vis spectroscopic titration was done in triplicate and the error bars were generated by Kaleidagraph. The spectral changes were linear up to [2HP]/[LOXL2] = 0.95 and reached a plateau at ≥ [2HP]/[LOXL2] = 1.0. (**C**) Structures of the LTQ cofactor (left) and LTQ−2HP model compound, **1** (right). A numbering system of carbons of the LTQ cofactor in the enzyme [4] is applied to the model compound, **1**.

The PH−derivatized LOXL2 was yellow and exhibited a UV−vis spectrum with λ_max_ of 445 nm (Figure 2A) that was superimposable to that of the model compound mimicking the LTQ−PH adduct, which was predominantly in the azo form in the hydrazone−azo tautomeric equilibrium (Figure S1) [21]. When LOXL2 was incubated with 2HP, the solution turned magenta−pink instead of yellow, and the final spectrum exhibited a λ_max_ of 531 nm, which was ~90 nm red−shifted when compared to that of LOXL2−PH (Figure 2A). The UV−vis spectra of LOXL2 before and after titration with 2HP are shown in Figure 2B. An increase in absorbance at 531 nm was observed when a semiquantitative amount of 2HP was added sequentially to protein samples at pH 8 (Figure 2B, left panel). The spectral change at 531 nm saturated around [LOXL2]/[2HP] = 0.95 (Figure 2B, right panel), indicating that LOXL2 contains a nearly stoichiometric amount (~95%) of the LTQ cofactor. To−date, this is the highest amount of LTQ detected in LOXL2.

### 2.2. UV−Vis Spectroscopic pK_a_ Determination of the LTQ−2HP Model Compound ***1***

**Figure 3:**
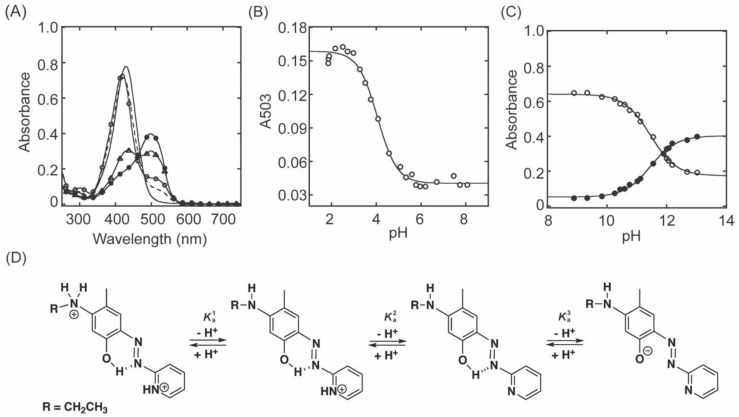
UV−vis spectroscopic titration of p*K*_a_s of **1**. (**A**) UV−vis spectra of **1** at pH 1.86 (–○–), pH 4.08 (−−−−), pH 8.07 (⎯⎯⎯), pH 11.90 (⎯△⎯) and pH 13.01 (⎯●⎯). [1] = 0.02 mM (**B**) The acidic p*K*_a_ of **1** was determined at 503 nm (⎯○⎯). The absorbance at 503 nm (A503) decreases between pH 2–6. The non−linear curve fit was performed using the equation, $A_{503}=\left\{\left(\in_{503}^{AH}\left[H^{+}\right]+\in_{503}^{A}K_{a}^{1}\right)/\left(\left[H^{+}\right]+K_{a}^{1}\right)\right\}{\left[A\right]}_{T}.$ (**C**) The basic p*K*_a_ of **1** was determined at 428 nm (⎯○⎯) and 500 nm (⎯●⎯).Between pH 8 to 13, the absorbance 428 nm (A428) decreases but the absorbance at 500 nm (A500) increases. The non−linear curve fit was performed using the following equations, $A_{428}=\left\{\left(\in_{428}^{A}\left[H^{+}\right]+\in_{428}^{A-}K_{a}^{2}\right)/\left(\left[H^{+}\right]+K_{a}^{2}\right)\right\}{\left[A\right]}_{T}$ and $A_{500}=\left\{\left(\in_{500}^{A}\left[H^{+}\right]+\in_{500}^{A-}K_{a}^{2}\right)/\left(\left[H^{+}\right]+K_{a}^{2}\right)\right\}{\left[A\right]}_{T}$. AH is the monoprotonated, A is the neutral and A^−^ is the monoanion species of **1**. (**D**) The p*K*_a_ assignment of **1**.

Το understand the nature of the 531 nm species formed in 2HP−inhibited LOXL2 (Figure 2A,B), a model compound mimicking the LTQ−2HP adduct (**1**, Figure 2C) was synthesized. Model **1** exhibits a UV−vis absorption spectrum with a λ_max_ at 428 nm at a physiological pH analogous to the model compound mimicking the 2HP adduct of TPQ in CAOs, TPQ−2HP (Figure S1A). The p*K*_a_s of TPQ−2HP were previously determined by UV−vis spectroscopic pH titration as p*K*_a_^1^ = 2.56 ± 0.08, p*K*_a_^2^ = 5.92 ± 0.05, and p*K*_a_^3^ = 13.1 ± 0.1 [26]. The 430 nm species of TPQ−2HP observed at physiological pH was assigned as a mono anion (Figure S1A,C). Similarly, the p*K*_a_s of **1** (Figure 3D) were determined to be p*K*_a_^2^ = 4.05 ± 0.05 (Figure 3A,B) and p*K*_a_^3^ = 11.46 ± 0.03 (Figure 3A,C) by UV−vis spectroscopic pH titration. The former was assigned to the pyridine nitrogen and the latter was assigned to the 4−hydroxyl group (Figure 3D) based on our analysis of p*K*_a_s of model compounds mimicking LTQ−PH [21] and TPQ−2HP [26] (Figure S1). Although TPQ−2HP and **1** both have λ_max_ at 430 nm at physiological pH, their protonation states are different (e.g., TPQ−2HP is a mono anion and **1** is a neutral species). We were not able to experimentally determine the p*K*_a_ of the alkylated aniline nitrogen (p*K*_a_^1^) of **1**, as it is lower than pH 1.5. The deprotonation of the 4−hydroxyl group (p*K*_a_^3^) of **1** caused a ~70 nm red−shift of λ_max_ (500 nm), but at ~30 nm it is blue−shifted (Figure 3A) when compared to the 531 nm species observed for 2HP−inhibited LOXL2 (Figure 2A,B). The p*K*_a_ of the 4−hydroxyl group (p*K*_a_^3^) of **1** was very similar to that of LTQ−PH (11.90 ± 0.04) (Figure S1B), but ~2 units smaller than that (13.1 ± 0.1) were determined for TPQ−2HP (Figure S1A). For TPQ−2HP, the formation of a double−negatively charged species is most likely accountable for the higher p*K*_a_ value (Figure S1A).

### 2.3. Effect of Cu^2+^ and Zn^2+^ on UV−Vis Spectrum of ***1*** at pH 8.0

**Figure 4:**
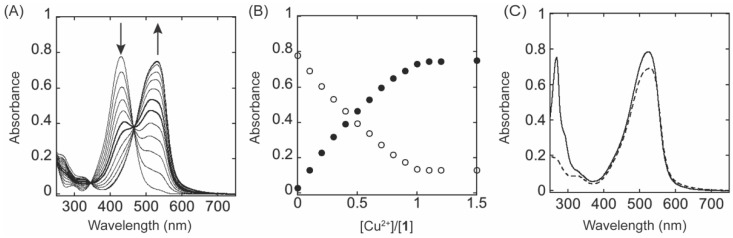
Cu^2+^ causes ~100 nm red−shift in λ_max_ of **1** at pH 8.0. (**A**) UV−vis spectral changes of **1** upon titration with Cu^2+^. [1] = 0.02 mgmt. (**B**) The absorbance changes at 430 nm (○) and 526 nm (●) saturated at [Cu^2+^]/[1] = 1. (**C**) The comparison of the final spectrum of **1** titrated with Cu^2+^ (–––) and **1** mixed with an equal amount of dichloro(1, 10−phenanthroline)Cu(II) (----).

The UV−vis spectroscopic titration of **1** with Cu^2+^ was performed at pH 8.0. The sequential addition of 0.1 molar equivalents of Cu^2+^ caused the decrease in absorbance at 430 nm and the increase in absorbance at 526 nm with isosbestic points at 342 and 466 nm (Figure 4A). The changes in absorbance at 430 nm and 526 nm were plotted against the molar ratio of [Cu^2+^]:[1]. Cu^2+^ binds to **1** forming a 1:1 complex, and no further increase in absorbance at 526 nm was observed by the addition of excess Cu^2+^ to **1** (Figure 4B). The sequential addition of 0.1 molar equivalent of Zn^2+^ also caused the decrease in absorbance at 430 nm and the increase in absorbance at 526 nm. In addition, an increase at 502 nm was detected (Figure S2A). These spectral changes were also saturated at one equivalent of Zn^2+^ over **1** (Figure S2B). The absorbance at 526 nm is expected to be the formation of a complex similar to **1** and Cu^2+^ but we need further study to identify the species at 502 nm.

**Figure 5:**
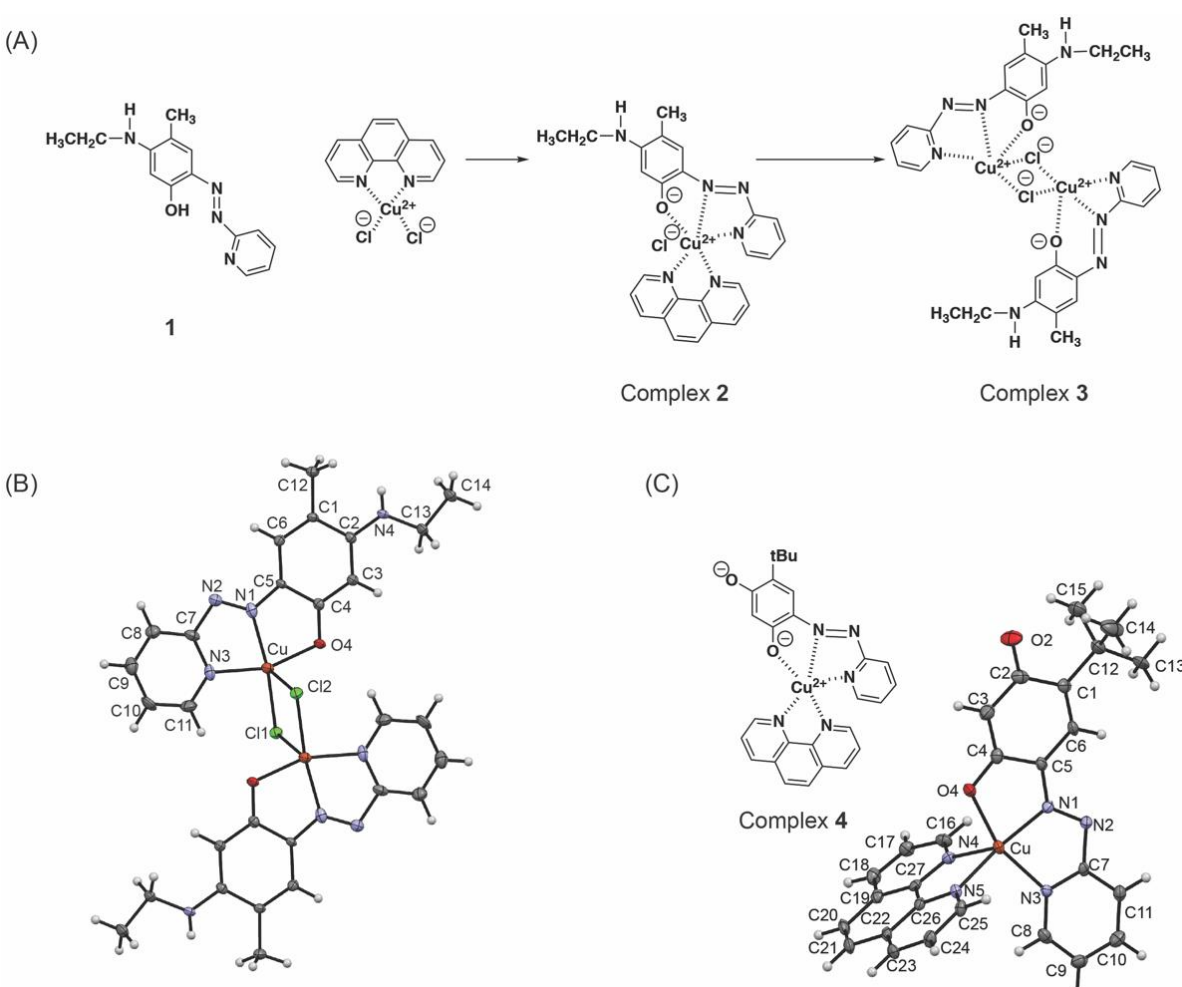
Structures of model compounds in this study (**A**) Complex **2** was generated in methanol solution by mixing equimolar amounts of **1** and dichloro(1,10−phenanthroline)Cu(II). Crystallization of Complex **2** by slow evaporation of methanol yielded Complex **3**. (**B**) An ORTEP view of the X−ray crystal structure of Complex **3 ** [30]. (**C**) Structure of Complex **4** (left) and an ORTEP view of Complex **4** (right) [29]. Displacement ellipsoids are shown at the 50% probability level. Symmetry code: −x+1, −y, −z. Copper: in copper, carbon: in black, nitrogen: in blue, oxygen: in red, chloride: in green, hydrogen: in white. The atom labeling system here is aligned with those used for LTQ and TPQ cofactor labeling in proteins [4,31]. Although Complex **3** is symmetrical, two Cl atoms are labeled Cl1 and Cl2 to address the coordination geometry of the Cu(II) center.

Since we observed a 502 nm species associated with deprotonation of 4−hydroxyl group of **1** under UV−vis spectroscopic pH titration (Figure 3A), it is possible that the 502 nm species is the monoanion of ligand **1**. Zn^2+^ may act as a tridentate ligand with a weaker interaction with 4−hydroxyl group of **1**, following the Irving−Williams series [27,28] and that is in equilibrium with the tetracoordinated 526 nm species. When we added an equimolar amount of dichloro(1,10−phenanthroline)Cu(II) to the solution of **1**, we obtained the UV−vis spectrum with λ_max_ at 526 nm (Figure 4C, solid line) which is very similar to that of the final titration product of **1** with Cu(II) (Figure 4C, dashed line). The additional feature below 300 nm originates from 1,10−phenanthroline−Cu(II), suggesting the formation of Complex **2** (1:1 complex of **1** and 1,10−phenanthroline−Cu(II)) (Figure 5). Dichloro(1,10−phenanthroline)Cu(II) was previously used for TPQ−2HP in a recombinant CAO from *Escherichia coli*, ECAO, to mimic the coordination environment of the active site Cu^2+^ [29].

### 2.4. X−ray Crystallography of the Complex of ***1*** and Cu(II), Complex ***3***

Upon crystallization of Complex **2** from anhydrous methanol, we obtained small dark red crystals of Complex **3** (Figure 5A). The ORTEP representation of the crystal structure of Complex **3** with atomic numbering scheme is shown in Figure 5B. The crystal structure of Complex **3** revealed a centrosymmetric Cu(II) dimer with a double axial−equatorial chlorido bridge, where another molecule of **1** replaced the original 1,10−phenanthroline ligand of Cu(II) [30]. This was not expected as we previously obtained crystals of TPQ−2HP complex with 1,10−phenanthroline−Cu(II) in Complex **4** (Figure 5C) [29]. In Complex **3**, each Cu(II) is 5−coordinate where N1, N3, O4 of **1** and one Cl of the chlorido bridge comprise the rough plane and the axial position is occupied by another Cl of the bridge. The axial Cu−Cl1 bond (2.639[1] Å) is longer than the equatorial Cu−Cl2 bond (2.309[9] Å) (Table 1), suggesting that it is the Jahn−Teller axis [32]. The Addison parameter (τ) was calculated as 0.004 by using the formula,

τ = (β − α)/60(1)

(α = 160.1[4], β = 160.3[8]; these are the largest valence angles). This indicates that the Cu(II) coordination geometry of Complex **3** is nearly square pyramidal as τ = 0 is the ideal square pyramidal geometry and τ = 1 is the ideal trigonal bipyramidal geometry [32]. In Complex **4**, Cu(II) is also 5−coordinate with N1, N3, O4 of TPQ−2HP, and N4 and N5 of 1,10−phenanthroline. Cu−N5 (2.229[4] Å) is the Jahn−Teller axis (Table 1), where τ was calculated as 0.080 (α = 160.3[9], β = 165.1[1]), suggesting that the Cu(II) coordination geometry of Complex **4** is more distorted from the ideal square pyramid than that of Complex **3** due to the bite angle of 1,10−phenanthroline. The dimer formation is most likely to cancel the total of +1 charge (+2 of Cu(II) and −1 of 4−oxoanion of **1**) where the shorter Cu−Cl provides −1 charge to each monomer. In contrast, in Complex **4**, TPQ−2HP is a dianion (discussed under *2.2*) to cancel the +2 charge of Cu(II). In LOXL2, two His residues of the Cu^2+^−binding site (His626−X−His628−X−His630) are most likely to replace the two Cl ligands of Cu(II) in Complex **3** to provide the square pyramidal Cu^2+^−coordination geometry and the +1 charge of the complex should be cancelled by a water molecule associating with Cu^2+^.

### 2.5. Electron Paramagnetic Resonance (EPR) Spectroscopy of Complex ***3*** in Solid State and in Solution

**Figure 6:**
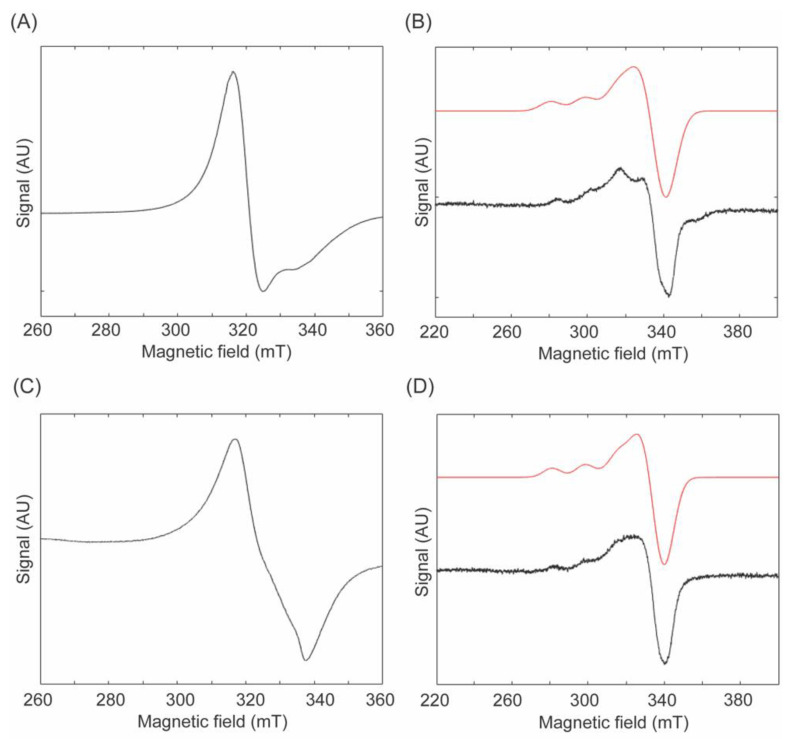
EPR spectroscopy at 77K. (**A**) Complex **3** in solid state (**B**) Complex **3** in DMSO (**C**) Complex **4** in solid state (**D**) Complex **4** in DMSO, In black: experimental spectra; in red: simulated spectra.

Due to the precipitation of protein at liquid N_2_ temperature, it was not possible to obtain EPR spectrum of 2HP−inhibited LOXL2. Since we obtained the crystal structure of Complex **3** rather than Complex **2** (Figure 5A,B) [30], we conducted comparative EPR spectroscopy of Complex **3** and Complex **4** (Figure 5B,C) both in a solid state and in solution (DMSO) to define the coordination geometry and electronic structure of Complex **3** and examine the effect of double chlorido bridges as opposed to 1,10−phenanthroline ligand (two Ns). Figure 6 shows the experimental spectra of Complex **3** and Complex **4** in a solid state as well an overlay between experimental (in black) and simulated (in red) data of 2 mM solutions of **3** and **4** in DMSO. The solid−state spectra display broad, largely isotropic signals centered at g = 2.148 and g = 2.117 for Complex **3** and Complex **4**, respectively. Magnetic interactions between separate Cu(II) centers in the solid state likely contribute to the unusual breadth of these signals (~50 mT), which obscures hyperfine features. In Complex **3**, these magnetic interactions are likely due to the close contact of the Cu(II) centers. Whereas the solid−state structure of Complex **4** reveals larger Cu−−Cu separations of ~8 Å, the 1,10−phenanthrene ligands in adjacent species show strong - stacking interactions. Such interactions have been previously shown to mediate Cu(II) exchange interactions even at long distances (~7–8 Å) [33]. In contrast to the relatively uninformative solid−state EPR spectra, the EPR spectra of Complex **3** and Complex **4** in DMSO show signals typical of a Cu(II) center in a square−pyramidal geometry. Simulation parameters for these spectra were summarized in Table 2. The g−value pattern, g_II_ > g_⊥_ > 2.0, is indicative of a dx^2^−y^2^ ground state in each complex, consistent with a square−pyramidal geometry [34]. In each case, the g_II_ is split due to hyperfine interactions with the Cu nucleus (*I* = 3/2). Collectively, the EPR data in DMSO provide strong evidence for very similar solution state structures for Complex **3** and Complex **4**.

### 2.6. Resonance Raman Spectroscopy 

**Figure 7:**
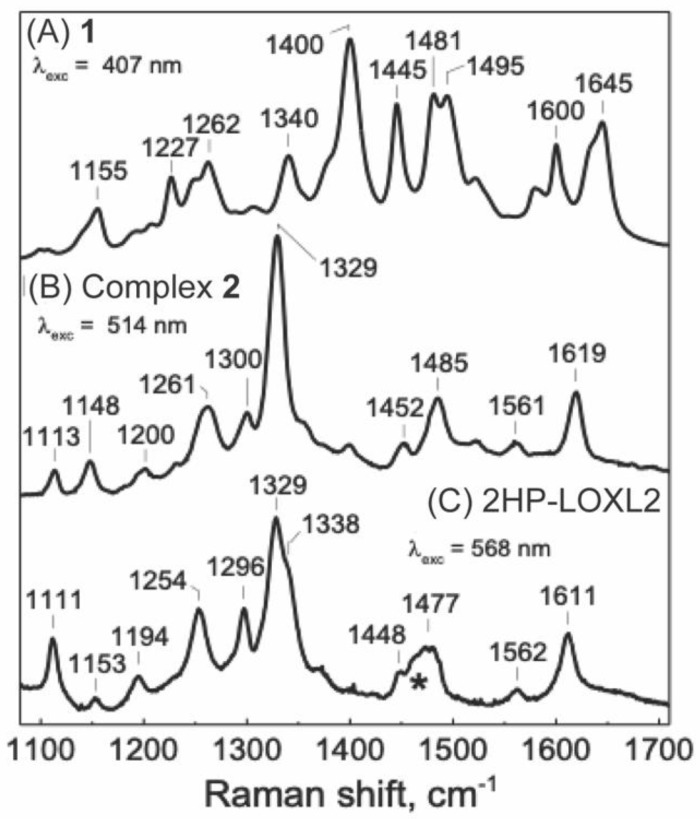
Room temperature RR spectra of **1** (**A**) and Complex **2** (**B**), and 2HP−inhibited LOXL2, 2HP−LOXL2 (**C**); the asterisk (*) denotes the Raman contribution of residual glycerol at 1470 cm^−1^.

In the resonance Raman (RR) spectroscopy of CAOs, model compounds mimicking quinone cofactors and their derivatives have been used as a frame of reference. Comparative study of enzymes and model compounds have also been used to successfully identify TPQ and LTQ in CAOs and LOX, respectively [20,31,35,36]. Figure 7 compares the high−frequency region of the RR spectra of 2HP−inhibited LOXL2, **1**, and Complex **2**. In this 1100 to 1700 cm^−1^ region, the RR spectrum of 2HP−inhibited LOXL2 is remarkably similar to the spectrum of Complex **2**, with its most intense bands at 1329 cm^−1^, a single band above 1600 cm^−1^, and a set of five fingerprint bands between 1110 and 1300 cm^−1^. In contrast, the spectrum of **1** alone is composed of a greater number of RR bands with similar intensities in the 1400 to 1650 cm^−1^ region dominated by C=C and C=N stretching vibrations. These RR spectroscopy results support a direct interaction of the Cu^2+^ ion with the hydrazo−hydroxyl moiety in 2HP−inhibited LOXL2 as in Complex **2**. Comparing RR spectra obtained using other laser excitations does not affect these conclusions (Figure S3).

### 2.7. Titration of Active Site Cu^2+^ in LOXL2 by 4−(2−Pyridilazo) Resorcinol, PAR

**Figure 8:**
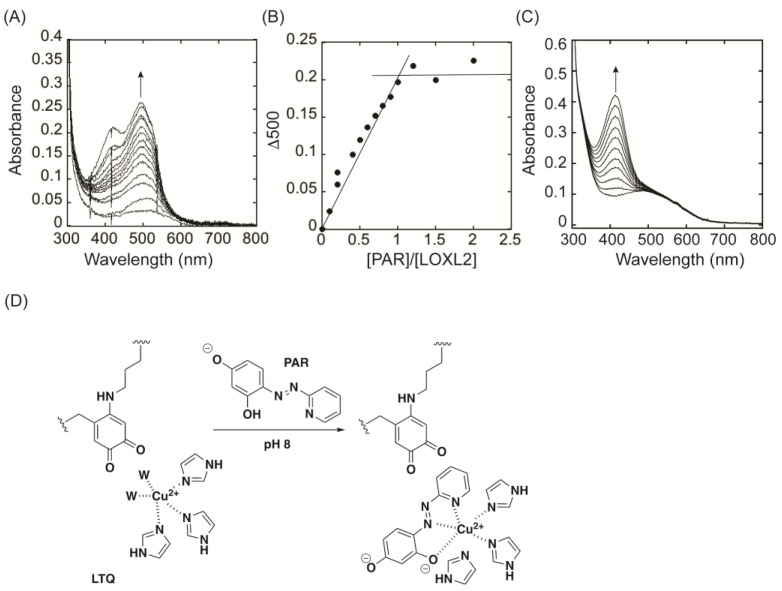
UV−vis spectroscopic titration of LOXL2 and AGAO with PAR. (**A**) UV−vis spectral changes observed upon titration of LOXL2 with substoichiometric amount (0.1 equivalent) of PAR. (**B**) Absorbance change at 500 nm (Δ500) was plotted against the molar ratio of [PAR]/[LOXL2]. The increase in absorbance at 500 nm saturates at [PAR]/[LOXL2] = 1.0. (**C**) A constant increase at 413 nm (free PAR) was observed but no increase in absorbance at 500 nm was observed upon titration of WT−AGAO with sub-stoichiometric amount (0.1 equivalent) of PAR. (**D**) Ligation of PAR to the active site Cu^2+^ lowers the p*K*_a_ of the 3−hydroxy group to generate the dianion (500 nm).

Interestingly, the azo form of LTQ−2HP contains the 4−(2−pyridilazo)resorcinol (PAR) moiety that is known to be a chelator for a variety of divalent cations [37,38]. In order to assess the solvent accessibility to the active site Cu^2+^ of LOXL2, UV−vis spectroscopic titration of the active site Cu^2+^ with PAR was conducted at pH 8 (Figure 8A). Upon titration with substoichiometric amounts (0.1 equivalent) of PAR over LOXL2, we observed the formation of the dianionic form of PAR at 500 nm due to the ligation of PAR to the active site Cu^2+^ (Figure 8D) (p*K*_a_s of PAR: pyridine N = 3.03 ± 0.17, para−OH = 5.50 ± 0.07, ortho−OH = 11.99 ± 0.05) [37] and the increase in absorbance at 500 nm reached a plateau at the molar ratio of [PAR]/[LOXL2] = 1 (Figure 8B). These results complement the Cu^2+^ content that was determined by Inductively coupled plasma (ICP) in this study (Table 3) as well as the nearly stoichiometric amount of the LTQ cofactor that was determined by UV−vis spectroscopic titration (Figure 2B). The monoanionic PAR absorbs at 413 nm and the excess of PAR (free form) was detected as an increase in absorbance at 413 nm after the spectral change at 500 nm plateaued (Figure 8A). These results suggest that the Cu^2+^ binding site of LOXL2 is solvent−exposed and that supports our proposed spatial arrangement of LTQ and Cu^2+^. 

### 2.8. Assessing the Importance of the Conserved His Residues in the Active Site of LOXL2

**Figure 9:**
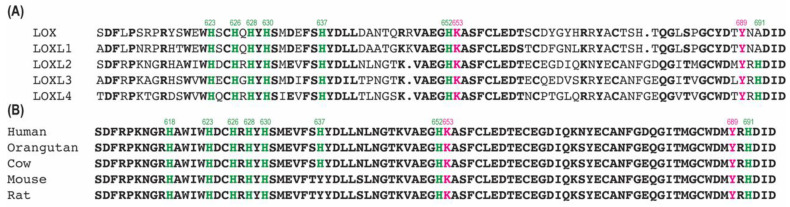
Conserved His residues (in green) in the active site of the LOX−family of proteins (**A**) and mammalian LOXL2s (**B**). The LTQ precursor residues are in pink. Residue numbers are those of human LOXL2.

Six His residues (His623, His626, His628, His630, His637, and His652) in the amine oxidase domain of LOXL2 are conserved among the human LOX−family of proteins (Figure 9A). We assessed the importance of these conserved His residues by site−directed mutagenesis. All six mutants are isolated from the culture media (Figure S4); therefore, these six His residues are not essential for protein secretion. As summarized in Table 3, the three His residues (H626Q, H628Q, H630Q) comprising the Cu^2+^−binding site are essential for LTQ biogenesis as expected. Interestingly, less than 50% of LTQ was detected both in His623Q and His652Q but His637 does not have a significant effect on LTQ biogenesis nor the catalytic activity of LOXL2. Although H637 is conserved in the human LOX−family of proteins (Figure 9A), it is not completely conserved in mammalian LOXL2s (Figure 9B), so it is most likely that H637 is not directly involved in LTQ biogenesis nor in the catalytic activity of LOXL2. Further study is necessary to define the role of His623 and His652 in LTQ biogenesis and catalytic activity.

## 3. Discussion

**Figure 10:**
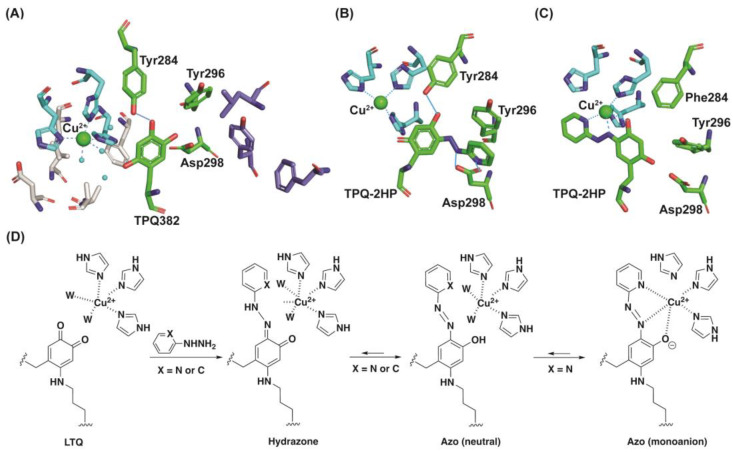
The active site environment of AGAO and LOXL2. (**A**) TPQ resides in between the Cu^2+^− and O_2_−binding sites (in light blue and in grey) and the substrate−binding site/entry channel (in dark blue). A hydrogen−bonding interaction between O4 (oxoanion) of TPQ and Tyr284 (in blue line) limits the mobility of TPQ [11]. (**B**) TPQ−2HP detected in WT−ECAO [26]. The residue numbering was edited to match with those of AGAO. (**C**) TPQ−2HP ligated on Cu^2+^ detected in Y284F−ECAO where Cu^2+^−ligation generates dianion of TPQ−2HP [29]. The residue numbering was edited to match with those of AGAO. (**D**) Formation of LTQ−2HP (azo) mono−anion by ligating to the active site Cu^2+^.The 2HP (X=N) adduct ligates to the active site Cu^2+^ but the PH (X=C) adduct does not. W: water molecule.

CAOs have two compartments in the active site (Figure 10A), one for amine oxidation (reduction of TPQ by substrate) and the other for Cu^2+^ binding and O_2_ binding (TPQ biogenesis and oxidation of substrate−reduced TPQ) [39]. The mobility of TPQ is carefully modulated in order to facilitate the catalytic oxidation of an amine, in particular by the hydrogen−bonding interaction of O4 of TPQ and the conserved Tyr (Tyr284 in AGAO, Tyr396 in ECAO) [11]. This hydrogen−bonding interaction confines the mobility of TPQ only to pivot in a wedge−shaped cavity in the active site to facilitate the Schiff base formation with a substrate amine. We have previously characterized TPQ−2HP in a CAO from *Escherichia coli* (ECAO). In WT−ECAO, TPQ−2HP was stabilized as the hydrazone form via hydrogen bonding interactions between the pyridine nitrogen of the 2HP moiety and the carboxyl group of Asp298 (active site base in AGAO numbering), and the quinone ring and pyridine ring were not coplanar (Figure 10B). The ligation of the TPQ−2HP adduct to the active site Cu^2+^ is suppressed via this hydrogen−bonding [26,29]. When these two hydrogen−bonding interactions were removed by site−directed mutagenesis, denaturation (pH 13) or heat (incubating at 60 °C), TPQ−2HP gained mobility, and the tautomerism shifted to the azo form (thermodynamically favored) and swung out of the wedge−like cavity surrounding TPQ to migrate onto the Cu^2+^−binding site to form TPQ−2HP−Cu^2+^ adduct (Figure 10C) [29].

In LOXL2, such hydrogen−bonding interactions between LTQ−2HP and active site residue(s) are clearly absent. The LTQ cofactor is a part of two peptides and not anticipated to have similar motional flexibility to TPQ. The results from this study suggest that the active site environment of LOXL2 is significantly different from that of CAOs. The detection of the dianionic form of PAR in LOXL2 (Figure 8A) and free PAR in AGAO (Figure 8C) indicates a substantial difference in solvent accessibility to the active site of these enzymes. Since we observed a direct formation of the 531 nm species in 2HP−inhibited LOXL2, LTQ is expected to be within 2.9 Å of the proposed Cu^2+^−binding site (Figure 10D) based on the structure of Complex **3** (Figure 5) and Cu^2+^−ligated TPQ−2HP in ECAO (Figure 10C).

## 4. Materials and Methods

### 4.1. Materials 

2−Aminopyridine, copper sulfate, 1,5−diaminopentane dihydrochloride (cadaverine), dichloro(1,10−phenanthroline) Cu(II), horseradish peroxidase (HRP), 2−hydrazinopyridine (2HP) dihydrochloride, and phenylhydrazine (PH) hydrochloride and 4−(2−pyridilazo)resorcinol (PAR) were purchased from SigmaAldrich (St. Louis, MO, USA). FreeStyle™ 293−F Expression System (FreeStyle™ 293−F cells, FreeStyle™ 293 Expression Medium, 293 fectin™ Transfection Reagent, Opti−MEM™I Reduced Serum Medium) and *N*−acetyl−3,7−dihydroxyphenoxazine (Amplex™ Red Reagent) were obtained from ThermoFisher Scientific (Lenexa, KS, USA).

### 4.2. Transient Transfection and LOXL2 Purification

FreeStyle™ 293F−cells were expanded in the FreeStyle™ 293 Expression Medium supplemented with 100 U/mL penicillin and 100 µg/mL streptomycin, whereas cell densities were maintained to ≥3 × 10^5^ cells/mL at 37 °C on an orbital shaker platform rotating at 125 rpm in a humidified atmosphere containing 8% CO_2_. The cap of the flask was kept loose at a quarter turn from snug to allow aeration. When the cell density in the suspension culture reached 1 × 10^6^ cells/mL, cells were transiently transfected with 1 mg/L of pcDNA3.1−LOXL2 [17] using 293fectin™ according to the manufacturer’s instructions. LOXL2 was purified following the method published previously [16]. 

### 4.3. Site−Directed Mutagenesis and Preparation of Recombinant LOXL2s

A series of His to Gln point mutants (H623Q, H626Q, H628Q, H630Q, H637Q, and H652Q) of LOXL2 were generated using pcDNA3.1−WT−LOXL2 as a template as previously described [17]. The primer pairs used to generate mutants (Table S1) were purchased from Eurofins Genomics (Louisville, KY, USA). Sequences were then validated by DNA sequencing at Genewiz (South Plainfield, NJ, USA). FreeStyle™ 293F−cells were expanded in the FreeStyle™ 293 Expression Medium supplemented with 100 U/mL penicillin and 100 µg/mL streptomycin, whereas cell densities were maintained to ≥3 × 10^5^ cells/mL at 37 °C on an orbital shaker platform rotating at 125 rpm in a humidified atmosphere containing 8% CO_2_. The cap of the flask was kept loose at a quarter turn from snug to allow aeration. When the cell density in the suspension culture reached 1 × 10^6^ cells/mL, cells were transiently transfected with 1 mg/L of pcDNA3.1−LOXL2 [17] using 293fectin™ according to the manufacturer’s instructions. LOXL2 was purified following the method published previously [16]. The copper contents of LOXL2s were determined by ICP−OES using a Thermo iCAP 7600 instrument in the Quantitative Bio−element Imaging Center (QBIC) at Northwestern University.

### 4.4. Amine Oxidase Activity Assay

The amine oxidase activity of LOXL2 was conducted on a BioTek Synergy H3 (Winooski, VT, USA) in the kinetics mode with the excitation wavelength at 544 nm and the emission wavelength set to 590 nm to monitor the formation of resorufin. A 54 μL of mixture of LOXL2, horseradish peroxidase (HRP) and Amplex Red was pre−equilibrated at 37 °C for 5 min. The reaction was initiated by the addition of 6 μL of cadaverine, and the standard substrate for in vitro LOX activity assay [18,40,41] to the reaction mixture and was monitored for 30 min. The final concentrations are: 40 nM LOXL2, 5 mU/mL HRP, 50 µM Amplex™ Red Reagent, 50 mM sodium borate buffer (pH 8.0). The slope of the increase in the fluorescence at 590 nm, and relative fluorescence units per minute (RFU min^−1^) in the linear region, were converted to a concentration of hydrogen peroxide per minute per concentration of LOXL2 ([H_2_O_2_] min^−1^μM^−1^). Data were taken in triplicates and the slopes of the linear range between 20–30 min were plotted against the concentration of cadaverine. The curve−fitting analysis was done on GraphPad Prism 9 (San Diego, CA, USA) and a non−linear regression curve−fitting with the Michaelis−Menten function (Equation (2)) was applied. *k*_cat_ was calculated by dividing *V_max_* (the maximum velocity) with the total LOXL2 concentration.

(2) $$v=\frac{V_{max}\left[cadaverine\right]}{K_{M}+\left[cadaverine\right]}\;\;\;\;\;\;\;\;\;\;\;$$

### 4.5. Preparation of the Wild−Type AGAO

The expression and purification of the precursor/apo form of the wild−type AGAO (apo−AGAO) was performed as previously described [10]. 

### 4.6. Synthesis of Model Compounds, Crystallization and X-ray Crystallography

Experimental details for synthesis of **1**, Complexes **2** and **3** and crystallization and X−ray crystallography of Complex **3** is described elsewhere [30].

### 4.7. UV−Vis Spectroscopy

UV−vis spectroscopy was performed on a Shimadzu UV2501 PC scanning UV/visible spectrophotometer with a Peltier temperature controller set at 25 ± 0.1 °C (path length of 1 cm). Data analysis was performed using Synergy Software Kaleidagraph v.4.5.4. (Reading, PA, USA).

#### 4.7.1. UV−Vis Spectroscopic LTQ Titration with 2HP in LOXL2

The UV−Vis spectroscopic titration of the LTQ cofactor in the mature LOXL2 was performed on an Agilent 8453 UV−Vis spectrophotometer. 2HP was added in a stepwise fashion of 0.1 molar equivalent to 100 μL of LOXL2 (350 μg) in 50 mM HEPES buffer pH 8.0. The reaction was incubated at 25 °C in a capped cuvette until no further spectral change occurred (15 min) after each addition of 2HP. The UV−vis absorbance was corrected for dilution effects.

#### 4.7.2. UV−Vis Spectroscopic p*K*_a_ Determination of LTQ−2HP Model (1)

A 10 μL aliquot of the stock solution ([1] = 2.0 mM in methanol) was diluted with 990 μL of 20 mM (*I* = 0.2 with NaCl) buffer of the desired pH, and UV−vis spectrum was recorded immediately after the dilution. The buffer solutions used and their pH, measured to ±0.01, were as follows: H_3_PO_4_ and NaH_2_PO_4_ for pH 0–3, CH_3_COOH and CH_3_COONa for pH 3−5.3, NaH_2_PO_4_ and Na_2_HPO_4_ for pH 5.6–7.8, NaHCO_3_ and Na_2_CO_3_ for pH 8.8−10.7, Na_2_HPO_4_ and Na_3_PO_4_ for pH 10.5–11.9, NaOH for pH > 12. 

#### 4.7.3. UV−Vis Spectroscopic Titration of **1** with Cu^2+^ at pH 8.0

A 10 μL aliquot of the stock solution ([1] = 2.0 mM in methanol) was diluted with 990 μL of 50 mM HEPES buffer, pH 8.0 (*I* = 0.2 with NaCl) in a quartz cell, giving a final concentration of [1] = 0.02 mM. A total of 10 μL aliquots of Cu^2+^ stock solution ([Cu^2+^] = 0.2 mM in milliQ H_2_O) was added to the solution of **1** to make the final concentration of Cu^2+^ between 0.002 mM–0.02 mM. The spectra were taken immediately after the dilution. Absorbance changes were corrected for dilution effects.

#### 4.7.4. UV−vis Spectroscopy of a Mixture of **1** and 1,10−Phenanthroline Cu(II), Complex **2**

A 10 μL aliquot of the stock solution ([1] = 2.0 mM in methanol) and 10 μL of dichloro(1,10−phenanthroline)Cu(II) (2.0 mM in methanol) was diluted with 980 μL of 50 mM HEPES buffer, pH 8.0 (*I* = 0.2 with NaCl) in a quartz cell, giving a final concentration of [1] = 0.02 mM and dichloro(1,10−phenantroline)Cu(II) = 0.02 mM. The spectrum was taken immediately after the dilution.

#### 4.7.5. UV−Vis Spectroscopic Titration of the Active Site Cu^2+^ with PAR in LOXL2

The quantitation of the active site Cu^2+^ was performed by UV−vis spectroscopically by stepwise additions of 0.1 equivalent molar of PAR to 100 µL of LOXL2 (200 μg) in 50 mM HEPES buffer at pH 8.0. Absorbance changes were corrected for dilution effects. 

#### 4.7.6. UV−Vis Spectroscopic Titration of the Active site Cu^2+^ with PAR in AGAO

The biogenesis of the TPQ cofactor was monitored by UV−vis spectrophotometrically in a reaction mixture containing 0.1 mM of apo−AGAO and 0.5 mM CuSO_4_ in 50 mM HEPES buffer at pH 7.2 (Buffer A) under air−saturated conditions [10]. The excess Cu^2+^ was removed by dialysis against Buffer A containing 50 mM of EDTA 3 times, followed by dialysis with Buffer A 3 times. The quantitation of the active site Cu^2+^ was then performed by stepwise additions of 0.1 equivalent molar of PAR to 100 µL of AGAO (0.065 mM) in Buffer A. UV−vis absorbance changes were corrected for dilution effects. 

### 4.8. EPR Spectroscopy

EPR spectra were acquired on a Bruker EMXplus spectrometer (Billerica, MA, USA) and samples were held at 77K using a finger dewar. For solid−state EPR spectroscopy, crystals of Complex **3** or Complex **4** were ground with a mortar and pestle to fine powders and placed in EPR tubes. For solution EPR spectroscopy, fine powders were dissolved in DMSO to make the final concentration of 2 mM and were frozen in liquid N_2_. Solution data were taken with a microwave frequency of 9.656 GHz and averaged over 15 scans, whereas solid−state samples were averaged over 5 scans. Additional acquisition parameters are in Table S2. Simulation of spectra and determination of g_II_ and A_II_ values (hyperfine) were performed by MatLAB 2021a [42] and EasySpin using the pepper algorithm with a Gaussian lineshape [43]. Values for g strain of each simulation can be found in Table S3.

### 4.9. Resonance Raman Spectroscopy 

Room−temperature resonance Raman (RR) spectra were obtained in a 90° geometry and a custom McPherson 2061/207 spectrograph with variable gratings, holographic long−pass edge filters from RazorEdge, Semrock, IDEX Health & Science, LLC (Rochester, NY, USA), and a LN−1100PB liquid−N_2_ cooled CCD camera from Princeton Instruments, (Trenton, NJ, USA). The 407−and 568−nm excitations were obtained from a Innova I302C Kr^+^ laser from Coherent Inc. (Santa Clara, CA, USA) and the 514−nm excitation from an Innova I90C Ar^+^ laser. Laser powers at the samples were maintained below 50 mW and the integrity of the samples throughout the experiments were confirmed by collecting UV−vis spectra of the sample inside the Raman capillaries before and after RR data collection. Frequencies were calibrated relative to indene and are accurate to ±1 cm^−1^. 2HP−inhibited LOXL2 and Complex **2** samples were both prepared at 100 μM concentration in a 5 mM HEPES buffer at pH 8.0 before taking RR spectra. 

### 4.10. Molecular Structure 

The molecular structures presented in this manuscript were prepared by PyMOL (The PyMOL Molecular Graphics System, Version 2.0 Schrödinger, LLC, New York, NY, USA; https://www.pymol.org) and Mercury [44].

## 5. Conclusions

In this study, we employed a recombinant LOXL2 containing a nearly stoichiometric amount of the LTQ cofactor and Cu^2+^ to conduct UV−vis, resonance Raman and EPR spectroscopy to assess the spatial arrangement of the LTQ cofactor and Cu^2+^ in the active site of the catalytically−competent LOXL2. The UV−vis spectroscopic titration of the LTQ cofactor with 2HP in the mature LOXL2 resulted in the direct formation of a species with 531 nm. Our study indicates that the 531 nm species in LOXL2 is LTQ−2HP ligated to the active site Cu^2+^. In contrast, TPQ−2HP detected in CAO was off−copper and it can ligate to the active site Cu^2+^ only when the hydrogen bonding interaction between the O4 of TPQ−2HP and the conserved Tyr396 was disrupted. Further, the UV−vis spectroscopic titration of the mature LOXL2 with PAR yielded the dianion of PAR (p*K*_a_^3^ = 11.99 ± 0.05) at pH 8.0, where ligation of PAR to the active site Cu^2+^ reduced the p*K*_a_ of 3−hydroxyl group of PAR by nearly five units. For AGAO, no formation of the dianion of PAR was observed. These results highlight the significant differences in the active site environment of LOXL2 and CAOs surrounding their quinone cofactors, LTQ and TPQ, respectively. The LTQ cofactor in the mature LOXL2 resides within 2.9 Å of the active site Cu^2+^ and both the LTQ and Cu^2+^−binding sites are solvent exposed.

## Figures and Tables

**Table 1:** Bond length (Å) and bond angles (°) of Complex **3** and Complex **4** at Cu(II) coordination.sites [29,30].

Complex 3 (Å)		Complex 4 (Å)		Complex 3 (°)		Complex 4 (°)	
O4−Cu(II)	1.9649[15]	O4−Cu(II)	1.986[4]	O4−Cu(II)−N3	160.67[7]	O4−Cu(II)−N3	160.3 [9]
N1−Cu(II)	1.9569[19]	N1−Cu(II)	2.007[4]	N1−Cu(II)−Cl1	161.09[6]	N1−Cu(II)−N4	165.11[4]
N3−Cu(II)	1.9906[19]	N3−Cu(II)	1.933[4]	N1−Cu(II)−Cl2	108.48[6]	N1−Cu(II)−N5	115.8[9]
Cl1−Cu(II)	2.2995[6]	N4−Cu(II)	2.030[4]	N3−Cu(II)−Cl1	92.90[6]	N3−Cu(II)−N4	110.1[9]
Cl2−Cu(II)	2.6187[6]	N5−Cu(II)	2.229[4]	N3−Cu(II)−Cl12	99.80[6]	N3−Cu(II)−N5	98.4[1]

**Table 2:** EPR simulation parameters for Complex **3** and Complex **4** in DMSO.

Compound	*g*_‖_	*A*_‖_ (MHz)	*g*_⊥_
Complex **3**	2.246	553.3	2.073
Complex **4**	2.248	540.6	2.077

**Table 3:** Properties and kinetic parameters of LOXL2 in cadaverine oxidation.

LOXL2 (0.03 μM)	LTQ (%)	Cu^2+^ (%)	*k*_cat_ (min^−1^)	*K*_m_ (mM)	*k*_cat_/*K*_m_ (min^−1^mM^−1^)	Relative *k*_cat_/*K*_m_ (%)
WT	100	0.030 (100)	9.28 ± 0.21	0.92 ± 0.07	10.09 ± 0.80	100
H623Q	<50	0.029 (97)	1.28 ± 0.15	0.06 ± 0.22	2.13 ± 0.82	21
H626Q	0	0.006 (20)	0	−	−	−
H628Q	0	0.004 (13)	0	−	−	−
H630Q	0	0.011 (37)	0	−	−	−
H637Q	90	N.D.	3.51 ± 0.26	0.48 ± 0.12	7.31 ± 1.91	72
H652Q	<50	0.016 (53)	3.06 ± 0.14	0.78 ± 0.10	3.92 ± 0.53	39

## Data Availability

Data may be available upon request.

## Supplementary Materials

The following supporting information can be downloaded at: https://www.mdpi.com/article/10.3390/ijms232213966/s1.
