# Supplementary material for: Insight into the Spatial Arrangement of the Lysine Tyrosylquinone and Cu2+ in the Active Site of Lysyl Oxidase-like 2
Source: Int J Mol Sci. 2022 Nov 12;23(22):13966. doi: 10.3390/ijms232213966 (PMC9694262; doi:10.3390/ijms232213966)
Supplement: Supplementary file 1 [file ijms-23-13966-s001.zip › ijms-1969267-supplementary.pdf]

Supporting Information

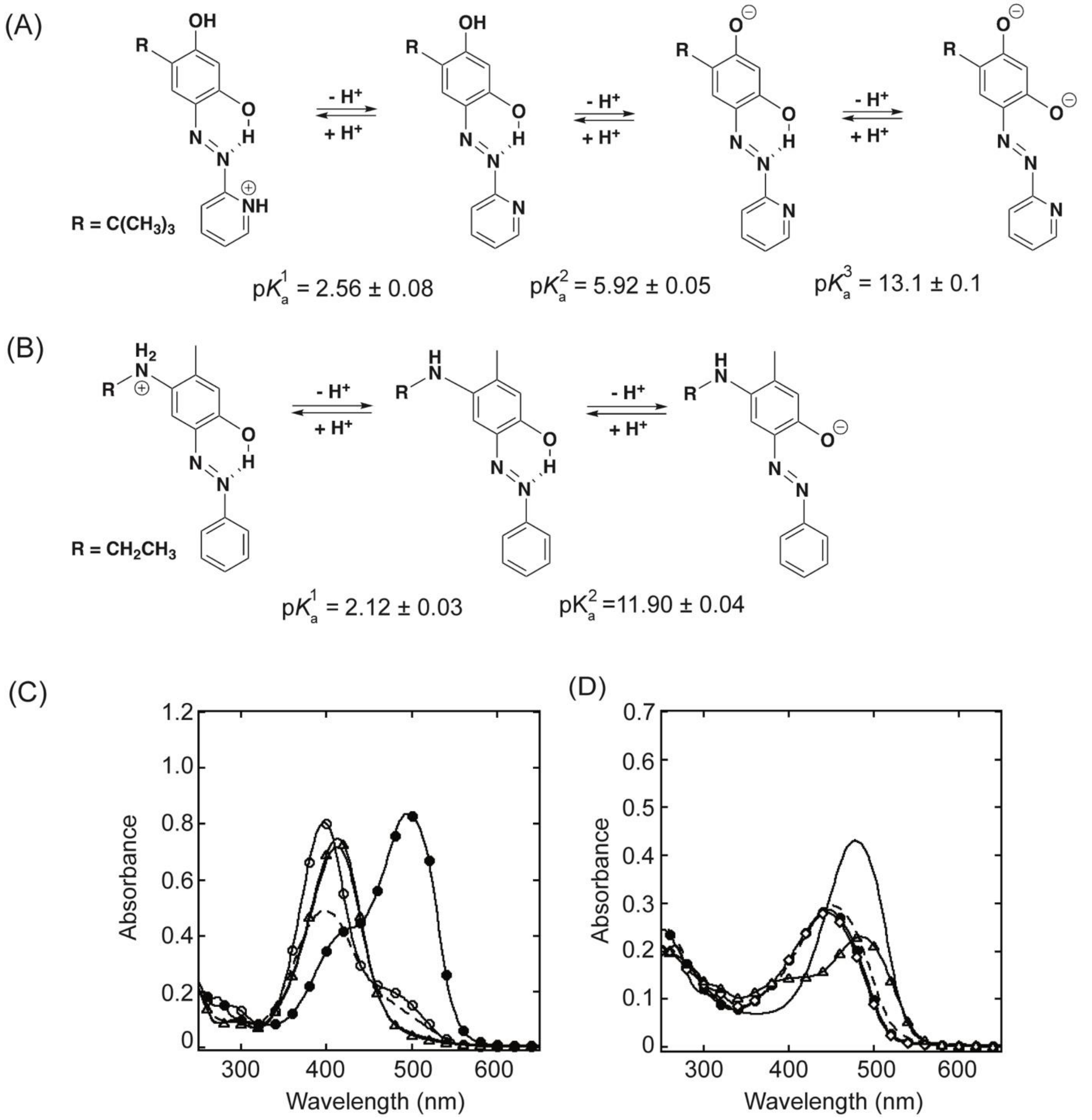

Figure S1. pK_as of TPQ-2HP (A) and LTQ-PH (B) determined by spectroscopic pH titration. C) UV-vis spectra of TPQ-2HP at pH -0.33 (–○–), pH 4.38 (----), pH 7.10 (——), pH 10.16 (–△–) and pH 13.18 (–●–) [1]. D) UV-vis spectra of LTQ-PH at pH 0.23 (–○–), pH 3.11 (----), pH 7.01 (——), pH 10.00 (–△–) and pH 13.43 (–●–)[2].

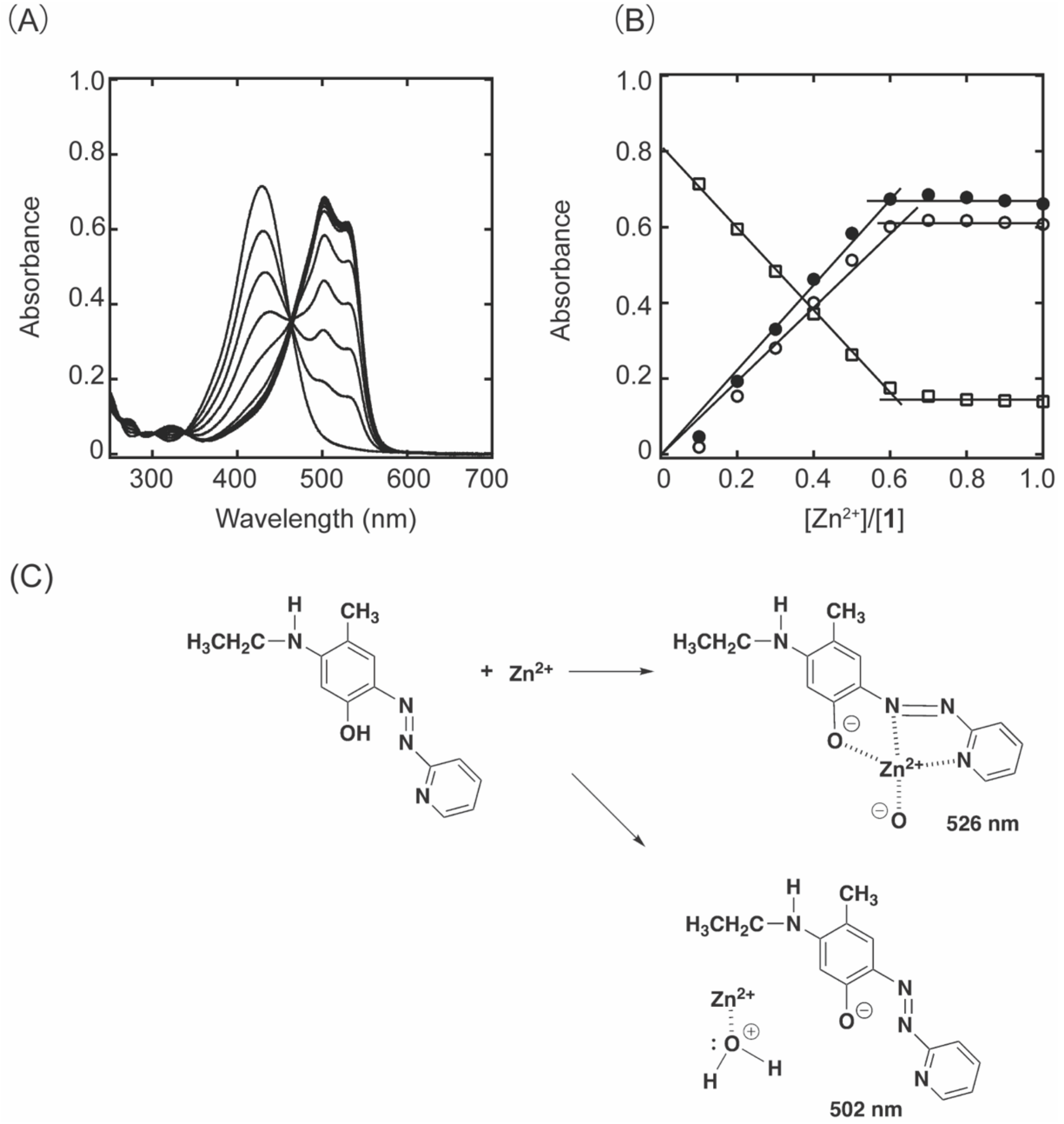

Figure S2. UV-vis spectroscopic titration of **1** with Zn^{2+} at pH 8.0. (A) UV-vis spectral changes observed during titration of **1** with Zn^{2+}. (B) Plot of absorbance changes at 430 (−☐−), 502 (−●−), 526 nm (−○−) versus the molar ratio of $[Zn^{2+}]/[\mathbf{1}]$. (C) Proposed mechanism for the formation of 502 and 526 nm species.

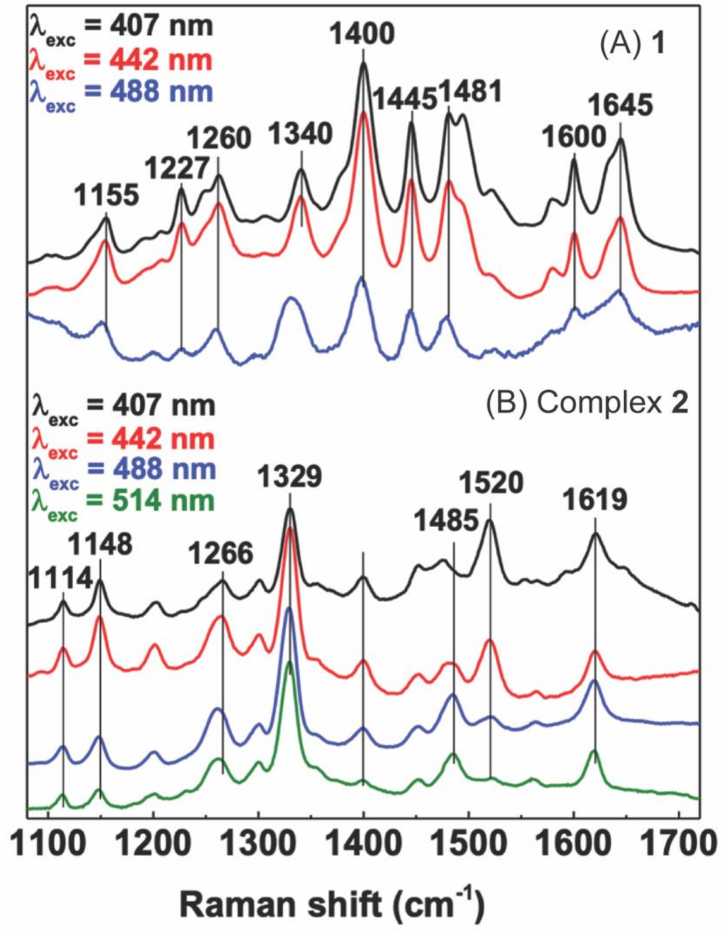

Figure S3. Room temperature RR spectra of **1** (A), Complex **2** (B) with varying excitation wavelength.

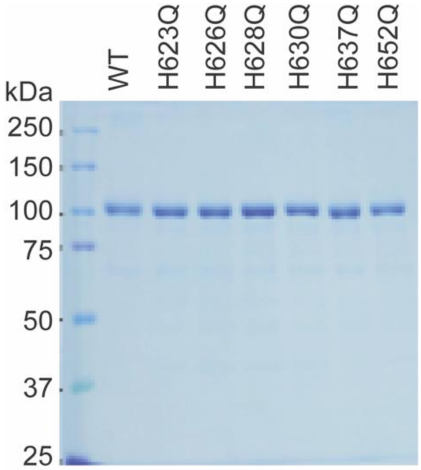

Figure S4. An SDS-PAGE analysis demonstrating that all mutants (H623Q, H626Q, H628Q, H630Q, H637Q, and H652Q) are secreted as WT-LOXL2.

Table S1. A set of primers used for site-directed mutagenesis.

Mutant	Primer	Sequence
H623Q	forward	CACGCGTGGATCTGGCAGGACTGTCACAGGCAC
	reverse	GTGCCTGTGACAGTCCTGCCAGATCCACGCGTG
H626Q	forward	GATCTGGCACGACTGTCAGAGGCACTACCACAGCATG
	reverse	CATGCTGTGGTAGTGCCTCTGACAGTCGTGCCAGATC
H628Q	forward	GCACGACTGTCACAGGCAGTACCACAGCATGGAGGTG
	reverse	CACCTCCATGCTGTGGTACTGCCTGTGACAGTCGTGC
H630Q	forward	CTGTCACAGGCACTACCAGAGCATGGAGGTGTTCACC
	reverse	GGTGAACACCTCCATGCTCTGGTAGTGCCTGTGACAG
H637Q	forward	ATGGAGGTGTTCACCCAGTATGACCTGCTGAAC
	reverse	GTTCAGCAGGTCATACTGGGTGAACACCTCCAT
H652Q	forward	AAGGTGGCAGAGGGCCAGAAGGCCAGCTTCTGC
	reverse	GCAGAAGCTGGCCTTCTGGCCCTCTGCCACCTT

Table S2. Acquisition parameters for EPR spectroscopy

Compound		Power (mW)	Power Attenuation (dB)	Field Center (G)	Sweep Width (G)
Complex **3**	solid state	2	20	3100	1000.1
Complex **4**	solid state	2	20	3100	1000.1
Complex **3**	in DMSO	2	20	2200	1800
Complex **4**	in DMSO	2	20	2200	1800

Table S3. g strain values in DMSO

Compound	g strain
Complex **3**	0.092
Complex **4**	0.080

References

1. Mure, M.; Brown, D. E.; Saysell, C.; Rogers, M. S.; Wilmot, C. M.; Kurtis, C. R.; McPherson, M. J.; Phillips, S. E.; Knowles, P. F.; Dooley, D. M., Role of the interactions between the active site base and the substrate Schiff base in amine oxidase catalysis. Evidence from structural and spectroscopic studies of the 2-hydrazinopyridine adduct of Escherichia coli amine oxidase. *Biochemistry* **2005,** 44, (5), 1568-82.
2. Mure, M.; Wang, S. X.; Klinman, J. P., Synthesis and characterization of model compounds of the lysine tyrosyl quinone cofactor of lysyl oxidase. *J Am Chem Soc* **2003,** 125, (20), 6113-25.
